# Beyond nimodipine: advanced neuroprotection strategies for aneurysmal subarachnoid hemorrhage vasospasm and delayed cerebral ischemia

**DOI:** 10.1007/s10143-024-02543-5

**Published:** 2024-07-05

**Authors:** Sabino Luzzi, Pınar Kuru Bektaşoğlu, Yücel Doğruel, Abuzer Güngor

**Affiliations:** 1 Department of Clinical-Surgical, Diagnostic and Pediatric Sciences, University of Pavia, Pavia, Italy; 2 Neurosurgery Unit, Department of Surgical Sciences, Fondazione IRCCS Policlinico San Matteo, Pavia, Italy; 3 Department of Neurosurgery, University of Health Sciences, Fatih Sultan Mehmet Education and Research Hospital, İstanbul, Türkiye; 4 Department of Neurosurgery, Health Sciences University, Tepecik Training and Research Hospital, İzmir, Türkiye; 5 Faculty of Medicine, Department of Neurosurgery, Istinye University, İstanbul, Türkiye

**Keywords:** Calcium channel blockers, Cerebral vasospasm, Delayed cerebral ischemia, Nimodipine, Rho-kinase inhibitors, Subarachnoid hemorrhage

## Abstract

The clinical management of aneurysmal subarachnoid hemorrhage (SAH)-associated vasospasm remains a challenge in neurosurgical practice, with its prevention and treatment having a major impact on neurological outcome. While considered a mainstay, nimodipine is burdened by some non-negligible limitations that make it still a suboptimal candidate of pharmacotherapy for SAH. This narrative review aims to provide an update on the pharmacodynamics, pharmacokinetics, overall evidence, and strength of recommendation of nimodipine alternative drugs for aneurysmal SAH-associated vasospasm and delayed cerebral ischemia. A PRISMA literature search was performed in the PubMed/Medline, Web of Science, ClinicalTrials.gov, and PubChem databases using a combination of the MeSH terms “medical therapy,” “management,” “cerebral vasospasm,” “subarachnoid hemorrhage,” and “delayed cerebral ischemia.” Collected articles were reviewed for typology and relevance prior to final inclusion. A total of 346 articles were initially collected. The identification, screening, eligibility, and inclusion process resulted in the selection of 59 studies. Nicardipine and cilostazol, which have longer half-lives than nimodipine, had robust evidence of efficacy and safety. Eicosapentaenoic acid, dapsone and clazosentan showed a good balance between effectiveness and favorable pharmacokinetics. Combinations between different drug classes have been studied to a very limited extent. Nicardipine, cilostazol, Rho-kinase inhibitors, and clazosentan proved their better pharmacokinetic profiles compared with nimodipine without prejudice with effective and safe neuroprotective role. However, the number of trials conducted is significantly lower than for nimodipine. Aneurysmal SAH-associated vasospasm remains an area of ongoing preclinical and clinical research where the search for new drugs or associations is critical.

## Introduction

The clinical management of aneurysmal subarachnoid hemorrhage (SAH)-associated vasospasm remains a challenge in neurosurgical practice, with its prevention and treatment having a major impact on the neurological outcome of the patient. Angiographic and clinically symptomatic vasospasm has been reported to account for up to 70% and 30% of patients, respectively, while delayed cerebral ischemia (DCI) occurs in nearly 20% [1–4]. Both have known detrimental effects on the brain.

Oral nimodipine is the mainstay of pharmacotherapy of cerebral vasospasm and DCI, as it is associated with improved outcomes and reduced mortality in patients with SAH, with indiscriminate type 1 A evidence [5–11]. Based on this data, it is the only drug approved by the US Food and Drug Administration and recommended by the latest AHA/ASA guidelines [12].

However, nimodipine-induced hypotension is a serious concern, as blood pressure fluctuations have been associated with the development of focal deficits and worse outcomes [13, 14]. The short half-life, pharmacokinetic variability, drug-drug interactions, and risk of vasoplegia are further shortcomings [15–18].

In recent years, drugs other than nimodipine have shown better pharmacokinetic profiles, but their efficacy and safety are still unclear.

The narrative review reported here aims to provide a comprehensive update of the pharmacodynamics, pharmacokinetics, overall evidence, and strength of recommendation of nimodipine alternative drugs in the prevention and treatment of aneurysmal SAH-associated vasospasm and DCI.

## Materials & methods

A comprehensive PICO (Population, Intervention, Comparison, and Outcomes) framework literature search was performed in the PubMed/Medline, Web of Science, and ClinicalTrials.gov databases using the following combination of MeSH terms “medical therapy,” “management,” “cerebral vasospasm,” “aneurysmal subarachnoid hemorrhage” and “delayed cerebral ischemia.” Eligibility criteria were as follows: full-text articles in English relevant to neuroprotection strategies for aneurysmal SAH-associated vasospasm and DCI. Articles were screened for typology and relevance prior to final inclusion. Editorials, case reports, and letters to the editor were excluded. Data analysis focused particularly on drugs other than nimodipine, and results were reported according to the PRISMA guidelines. The pharmacokinetic profiles of eligible drugs were obtained from PubChem and PubMed/Medline databases.

## Results

A total of 346 articles were initially identified. Twenty-seven were excluded as duplicates. Of the remaining articles, 207 were excluded as off-topic and 44 were eliminated for marginal relevance. The number of case reports, editorials, and letters to the editor was 3, 4, and 2, respectively. Fifty-nine studies were included in the review.

**Fig. 1:**
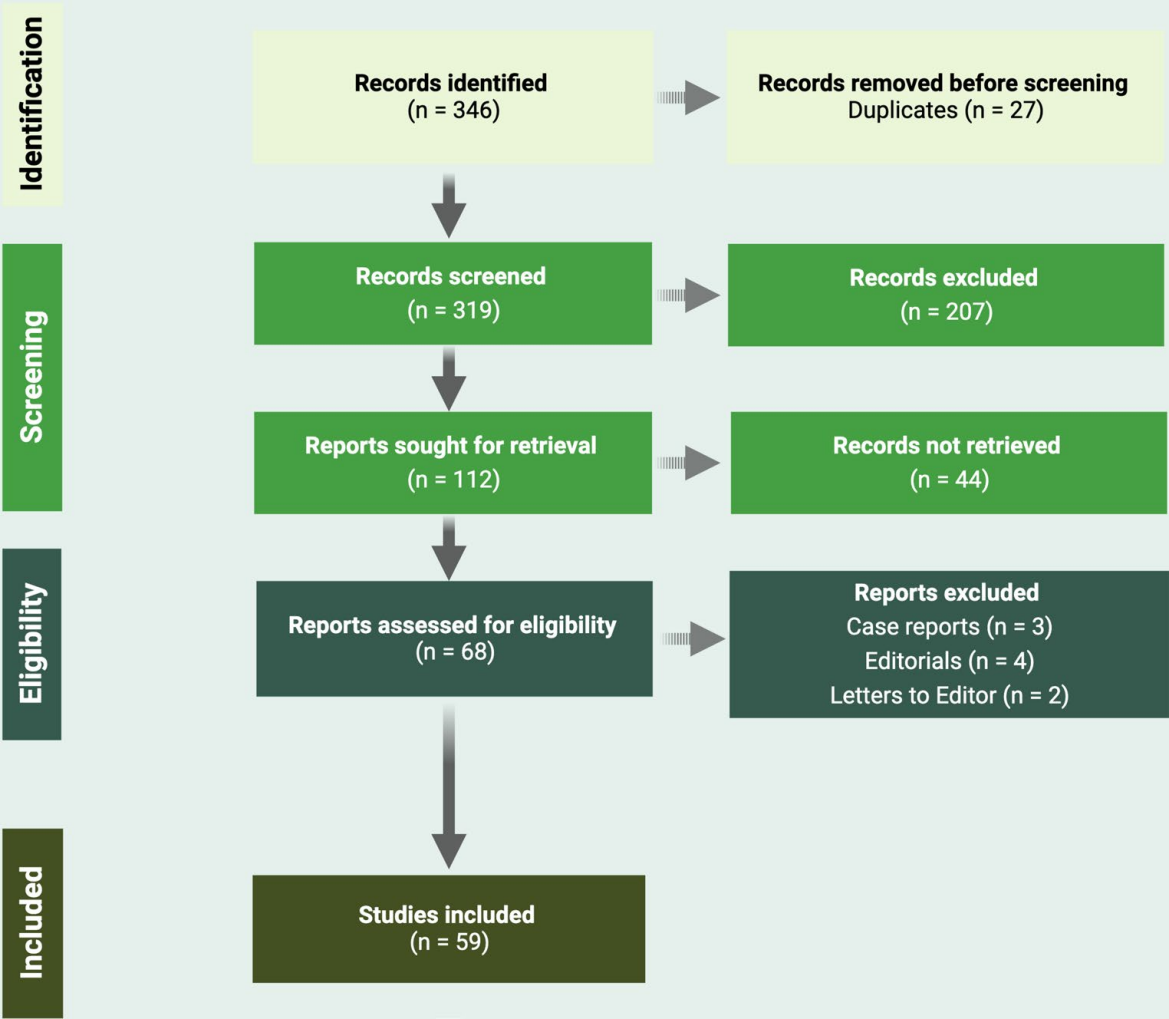
PRISMA flow-chart of the study

PRISMA flow chart and data derived from the identification, screening, eligibility, and inclusion process are summarized in Fig. 1.

### Nicardipine

Nicardipine is a dihydropyridine-type Ca(2+) channel blocker whose infusion efficacy in the treatment of vasospasm and DCI has been confirmed [19, 20]. In a Cochrane review, it was found to be not superior to nimodipine mainly because of insufficient data [5]. Conversely, its intraoperative intracisternal release proved highly effective in a phase II prospective randomized, double-blind trial [21].

### Verapamil

Verapamil belongs to Ca(2+) channel blockers. A recent systematic review highlighted that there are no randomized trials to support the routine use of verapamil in vasospasm and DCI, although observational data suggest possible clinical but underestimated benefits [22]. On a cohort series of 75 procedures from Burdenko Institute of Neurosurgery, intra-arterial verapamil proved to be effective in patients in the initial stage of DCI [23]. Similar results were reported by Mao and co-workers [24]. Verapamil has been reported to be the most common first-line therapy against aneurysmal SAH-associated vasospasm and DCI in the United States [25]. It has mainly been used intra-arterially in high doses [23, 26, 27]. Notably, it has not been shown to compromise hemodynamic stability, although data on increased intracranial pressure are conflicting [26, 28].

### Albumin

Albumin has neuroprotective effects due to its antioxidant and anti-inflammatory properties [1]. Increased oncotic pressure reduces cerebral edema, improves neuronal survival, and preserves the integrity of the blood-brain barrier. In the Albumin in Subarachnoid Hemorrhage (ALISAH) trial, albumin was safe in patients with SAH, and improved outcomes were reported with no major complications [29]. Higher doses of albumin were associated with a dose-dependent reduction in the incidence of vasospasm, DCI, and stroke at 90 days [30]. However, there was also a dose-related increase in cardiac complications. The recent ASA/AHA guideline reported that volume expansion increases the rate of medical complications without improving overall outcomes or reducing DCI [12].

### Cilostazol

Cilostazol, a platelet aggregation inhibitor, prevents the formation of microthrombi. It also has a vasodilatory effect by inhibiting phosphodiesterase 3 and increasing intracellular cAMP [31]. In a multicenter, prospective, randomized study, the incidence of angiographic vasospasm was significantly lower in the cilostazol group than in the placebo group. However, no significant difference in clinical outcomes was reported [32]. In a meta-analysis, cilostazol was associated with a reduced risk of symptomatic vasospasm, cerebral infarction, and poor outcome [33]. Cilostazol appears to be a safe and promising agent; however, it is not routinely used in clinical practice. Further evidence is needed.

### Corticosteroids

Corticosteroids reduce brain swelling and may improve recovery after SAH due to their anti-inflammatory effects.

A randomized trial of high-dose methylprednisolone reported better functional outcomes in patients with cerebral vasospasm after SAH [34]. In some units, corticosteroids are the preferred treatment for SAH patients, although a Cochrane review reported that the benefits outweigh the harms [35]. Further evidence is needed to determine their clinical significance.

### Dapsone

Dapsone is a well-known antimicrobial agent with antioxidant, anti-excitotoxic, and anti-apoptotic activity [36]. It has been studied in a small group of patients with SAH, and promising results in the prevention of DCI have been reported [37]. Anti-inflammatory and neuroprotective activity with glutamate receptor antagonist effect may explain the mechanism of its action in vasospasm.

### Omega-3 polyunsaturated fatty acids

The sphingosylphosphorylcholine-Rho-kinase pathway is involved in vascular smooth muscle contraction and plays an important role in cerebral vasospasm after SAH [1]. Eicosapentaenoic acid and docosahexaenoic acid, both omega-3 polyunsaturated fatty acids, have been shown to be effective in the clinical management of cerebral vasospasm after SAH through their inhibition of Rho-kinase [38, 39]. A prospective, multicenter, randomized trial validated the efficacy of eicosapentaenoic acid [39].

### Clazosentan

Endothelin-1 is a vasoconstrictive peptide that is overproduced in SAH [1]. Clazosentan inhibits the binding of endothelin-1 to its receptor.

CONSCIOUS-1, a phase II study, reported a dose-dependent reduction in angiographic vasospasm [40]. CONSCIOUS-2, a prospective, double-blind, placebo-controlled phase III study, showed no clinical improvement with treatment [41]. On the contrary, significant side effects (hypotension, cerebral infarction, pulmonary edema, and anemia) were reported. Because of these findings, the CONSCIOUS-3 study was stopped early [42]. Similar results were reported in 2018 in a meta-analysis of randomized controlled trials completed until that time [43]. The general interest in clazosentan was revived in the last 2 years thanks to JapicCTI163369 and JapicCTI163368, two Japanese phase III trials that reported the efficacy of this endothelin receptor antagonist in the prevention of cerebral vasospasm, vasospasm-related DCI, and cerebral ischemic symptoms after aneurysmal SAH [44]. This initial evidence led to the first approval of clazosentan for clinical use in Japan [45].

A recent meta-analysis of 2778 patients concluded that clazosentan reduces vasospasm-related DCI and angiographic vasospasm but doesn’t seem to improve functional outcome or mortality [46]. A possible explanation of this discrepancy can lie in the very high rate of adverse events the authors recorded, causing worse outcome, in those cohorts where high doses of clazosentan were administered.

### TAK-044

Similar to clazosentan, TAK-044 is an endothelin receptor antagonist which has been reported to have a trend towards reduced DCI, but with no benefit in clinical outcomes and hypotension as a side effect [47].

### Erythropoietin

Cerebrovascular endothelia have erythropoietin receptors, and erythropoietin administration is thought to have neuroprotective properties. A randomized phase II study reported a lower incidence of severe vasospasm, reduced DCI, and improved outcome at discharge after erythropoietin administration [48]. In another study, increased brain tissue oxygen pressure was found to be significant [49]. The mechanism of action and clinical significance require further evidence.

### Fasudil

Fasudil is a Rho-kinase inhibitor with vasodilatory, anti-inflammatory, and antioxidant effects. The largest study of intra-arterial administration of fasudil hydrochloride and a meta-analysis both showed that this molecule significantly prevented vasospasm and DCI in patients with SAH, also improving clinical outcomes [50, 51]. Fasudil is currently the preferred drug for vasospasm prophylaxis in Japan [52].

Ye et al. reported that the addition of fasudil to nimodipine treatment improved clinical outcomes in patients with SAH [52].

### Unfractionated heparin and low-molecular-weight heparin

Heparin has been proposed as a candidate in the prevention of vasospasm because of its capability to neutralize oxyhemoglobin, decreased the level of transcription of endothelin-1, inhibition of binding to vessel wall selectins, and counteraction of inflammatory response [53]. A large double-blind, single-center clinical trial on 170 patients failed to demonstrate any advantages of the routine use of enoxaparin [54]. The results of another still ongoing open-label, single-center, randomized trial on an larger cohort of patients will definitely clarify the role of unfractionated heparin and low-molecular-weight heparin in preventing ischemic complications after SAH [55].

### Papaverine

Papaverine is a known potent vasodilator capable of improving cerebral blood flow. Its intra-arterial use in conjunction with transluminal balloon angioplasty has been studied, showing positive angiographic results in selective vessel narrowing [56]. However, the high rate of discrepancy between prompt reversal of arterial narrowing and neurological outcome made the efficacy of this drug for vasospasm treatment and DCI prevention very weak [56–59]. The time-limited effect of papaverine, the need for repeated infusions and the consequent risk of blood pressure fluctuations are drawbacks.

### Tissue plasminogen activator (tPA)

Intracisternal tissue plasminogen activator (tPA) release after aneurysm clipping is thought to reduce the incidence of vasospasm and DCI because of its fibrinolytic properties. A multicenter, randomized, blinded, placebo-controlled trial demonstrated efficacy only in thick subarachnoid clots with a 56% relative risk reduction of severe vasospasm [60]. A meta-analysis documented an absolute risk reduction of vasospasm and mortality of 14.4% and 4.5%, respectively [61]. The efficacy of tPA has also been confirmed with intrathecal administration [62–64]. However, the risk of infection and other adverse effects should be further investigated for this route of administration.

### Urokinase

Intrathecal urokinase infusion and cisternal irrigation have been shown to be effective in preventing symptomatic vasospasm and DCI [64–67]. The pharmacodynamics are the same as described for tPA, but the overall level of evidence consists mainly of retrospective case series.

### Magnesium

Magnesium blocks voltage-dependent calcium channels and causes vasodilation of cerebral arteries [1]. Magnesium also blocks glutamate release and exerts neuroprotective effects. However, the penetration of magnesium into the cerebrospinal fluid is low [68]. MASH, a phase II trial, showed positive results with intravenous magnesium in patients with SAH [69]. IMASH and MASH-2, two phase III trials, showed no significant clinical efficacy [68, 70]. There are also side effects of intravenous magnesium treatment, such as hypocalcemia, hypotension. The combined form of magnesium with oral nimodipine has also been studied and reported to have advantages in the clinical management of cerebral vasospasm after SAH [71]. However, there is still insufficient evidence to support the routine use of magnesium in patients with SAH.

### Milrinone

Milrinone is a phosphodiesterase 3 inhibitor that increases intracellular cAMP levels and causes vasodilation [1]. Milrinone also has anti-inflammatory effects. It is occasionally used intravenously in cases of refractory vasospasm unresponsive to other interventions and is administered intra-arterially in patients undergoing endovascular interventions for symptomatic vasospasm [1]. In the Montreal Neurological Hospital protocol study, intravenous milrinone infusion was well tolerated with good functional outcome [72]. Systemic hypotension and tachycardia are notable side effects. Retrospective case-control studies and systematic reviews are the primary level of evidence [73–75].

### Minocycline

Minocycline, a tetracycline antibiotic, is recognized for its additional anti-inflammatory effects within the central nervous system. These effects include the inhibition of microglia/macrophage phagocytic activity and activation, as well as the inhibition of matrix metalloproteinase (MMP). Furthermore, minocycline exhibits the capability of iron chelation and inhibits SAH-induced neuronal cell death [76–82]. MMP-9 levels are elevated in patients with SAH, and elevated MMP levels have been correlated with the risk of vasospasm [83].

Studies of minocycline have shown promising results in patients with acute ischemic stroke and intracranial hemorrhage with a good safety profile [84–89].

Conversely, a randomized, double-blind, controlled trial failed to demonstrate the efficacy on vasospasm [90].

### Statins

Statins upregulate endothelial nitric oxide synthase and increase nitric oxide biosynthesis, thereby improving cerebral vasomotor reactivity [1]. Statins also reduce glutamate-mediated excitotoxicity and control the inflammatory response. The phase III STASH trial reported that simvastatin showed no benefit in either acute or long-term treatment of patients with SAH [91]. A recent systematic review of statins concluded that they may significantly reduce the incidence of ischemic cerebrovascular events and improve functional prognosis in patients with SAH [92]. Another systematic review and meta-analysis of 13 randomized controlled trials concluded that statins significantly reduce the incidence of vasospasm, DCI, and mortality after SAH [93].

Nevertheless, the most recent AHA/ASA recommendations state that there is a lack of evidence to support statin therapy in patients with SAH to prevent vasospasm [12].

### Tirilazad

Tirilazad is a free radical scavenger and antioxidant and has neuroprotective effects [1]. It is a non-glucocorticoid, 21-aminosteroid that prevents lipid peroxidation. A Cochrane meta-analysis that included five double-blind, placebo-controlled trials definitively showed no significant clinical efficacy of tirilazad treatment in patients with cerebral vasospasm after SAH [94].

### Pharmacokinetic profiles of nimodipine alternative drugs

**Fig. 2:**
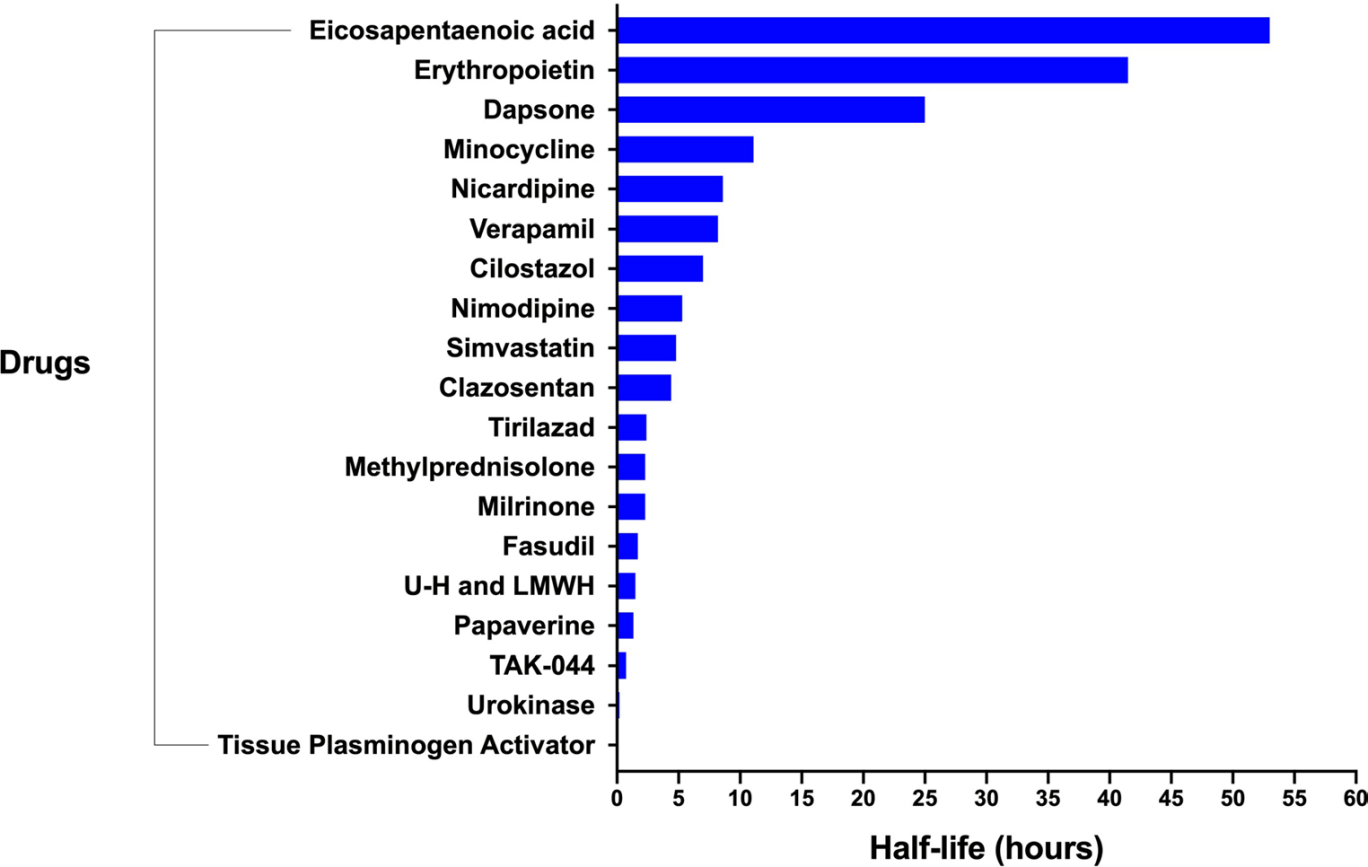
Bar graph showing the average half-life of the main nimodipine alternative drugs

Most drugs are administered orally. Time to peak concentration, protein binding and volume of distribution are highly variable. With the exception of corticosteroids, erythropoietin and heparin, all drugs are subject to hepatic metabolism by the cytochrome P-450 complex. Eicosapentaenoic acid, erythropoietin and dapsone have the longest half-lives, whereas the half-life of nimodipine has been reported to be between 1.7 and 9 h (Fig. 2).

The pharmacokinetic profile of nimodipine and the main nimodipine alternatives in cerebral vasospasm is summarized in Table 1.

**Table 1:** Pharmacokinetic profile of nimodipine and nimodipine alternative drugs in cerebral vasospasm and DCI

Drug	Parameters						
	Route of administration	Time to peak concentration (T-max) (hours)	Protein binding (%)	Distribution volume (L/kg)	Metabolism	Excretion Site (%)	Half-life (hours)
Nimodipine [95]	Oral* Intravenous Intraarterial	0.5-1	95	0.9–2.4	Hepatic cytochrome P-450 complex	Urine (99) Bile (1)	1.7-9
Nicardipine [96]	Oral	0.2-2	97	0.4	Hepatic cytochrome P-450 complex	Urine (30) Bile (70)	8.6
Verapamil [97]	Oral	1–2	94	0.3–0.5	Hepatic cytochrome P-450 complex	Urine (70) Bile (30)	4.5–12
Cilostazol [98]	Oral	3	95–98	2.7	Hepatic cytochrome P-450 complex	Urine (74) Bile (20)	11–13
Methylprednisolone [99]	Oral Intramuscular Intravenous	0.2–2.5	78	1.3	Adrenal Glands, Liver, Kidney	Urine (48) Bile (52)	2.3
Dapsone [100]	Oral	4	50–90	1.5–2.5	Hepatic cytochrome P-450 complex	Urine (80) Bile (20)	20–30
Eicosapentaenoic acid [101]	Oral	5	88	1.2	Systemic lipoxygenases and cyclooxygenase	NA	39–67
Clazosentan [102]	Intravenous	NA	99	1.3–3.2	Hepatic cytochrome P-450 complex	Urine (80) Bile (20)	1.8–2.6
TAK-044** [103, 104]	Intraarterial	0.1	NA	0.23	Hepatic	Urine (10) Bile (90)	0.5-1
Erythropoietin [105]	Subcutaneous Intravenous	5–24	NA	40-63.8	Kidney	Lymphatic system (95%) Urine (5%)	16–67***
Fasudil* [106]	Oral	0.3	NA	142.6	Hepatic cytochrome P-450 complex	NA	1.7
U-H and LMWH [107–109]	Subcutaneous Intravenous	2–4	67	4–7	Reticuloendothelial system of arterial and venous endothelium	Urine (98) Bile (1)	1.5
Papaverine [110]	Oral Intraarterial	1	90	3.1	Hepatic cytochrome P-450 complex	Urine (99) Bile (1)	0.5–2.2
Tissue Plasminogen Activator [111–114]	Intravenous	3	NA	8.1	Hepatic cytochrome P-450 complex	Urine (80) Bile (20)	0.08
Urokinase [115, 116]	Intravenous	NA	NA	11.5	Hepatic proteases	Urine (80) Bile (20)	0.2
Magnesium [117, 118]	Oral	2	20	13.6–49	NA	Urine (99) Bile (1)	1000
Milrinone [119, 120]	Intravenous	0.05	70	0.38	Hepatic cytochrome P-450 complex	Urine (99) Bile (1)	2.3
Minocycline [121–123]	Oral	1.9	76	67.5–115	Hepatic cytochrome P-450 complex	Urine (10) Bile (90)	11.1
Simvastatin [124–126]	Oral	1.3–2.4	95	NA	Hepatic cytochrome P-450 complex	Urine (40) Bile (60)	4.8
Tirilazad [127–129]	Oral	4–6	85**	4.8**	Hepatic cytochrome P-450 complex	Urine (5) Bile (95)	2.4**

*preferred, **pharmacokinetics determined on rats, ***subcutaneous administration, NA: not available, U-H: Unfractionated Heparin, LMWH: Low-Molecular-Weight Heparin

## Discussion

In this narrative review, the pharmacodynamics, pharmacokinetics, and overall level of evidence for nimodipine alternatives in the prevention and treatment of aneurysmal SAH-associated vasospasm and DCI are outlined.

The reported data also lead to a grading of the strength of the recommendations for the nimodipine alternative drugs, the rationale for which is to overcome some of the intrinsic shortcomings of nimodipine itself.

### Nimodipine pharmacodynamic, pharmacokinetic, strengths and weaknesses

The incidence of vasospasm in large series has been reported to be higher in medial and anterior circulation aneurysms [130–134], while lower rates have been observed in ruptured posterior cerebral artery aneurysms [135]. These findings have also been confirmed in the authors’ own series [136–145]. Nimodipine is the backbone in the prevention and treatment of vasospasm and ischemic related complications [11, 146, 147], it being the only medication approved by the US Food and Drug Administration and recommended by the AHA/ASA guidelines [12]. Although it is known to inhibit the influx of calcium ions through voltage-gated L-type calcium channels of vascular smooth muscles [148], this phenomenon is not held responsible for improving clinical outcome of SHA and DCI patients. Possible proposed mechanisms of action targeting vasospasm are instead increase in fibrinolytic activity, neuroprotection, and inhibition of cortical spreading ischemia [149–151]. There are a few doubts about the advantages of nimodipine oral administration in decreasing the induced blood pressure oscillations [5–7]. Conversely, possible significant rise in favorable outcome after intraventricular or cisternal administration has been ruled out by NEWTON and NEWTON-2 trials [152, 153].

Notwithstanding the type 1 A pieces of evidence, nimodipine is not free from pharmacokinetic and pharmacodynamic limitations lying in a short half-life, interpatient pharmacokinetic variability, potential for drug-drug interactions, induced hypotension, blood pressure fluctuations, risk of recalcitrant vasoplegia, and rare unresponsiveness [12, 154–156]. The detrimental effects of potential nimodipine-induced hypotension are also well known and the ASA/AHA guideline recommend even the nimodipine cessation till the correction of blood pressure in case of severe hypotension [12].

### Nimodipine pharmacokinetic variability

Nimodipine has been associated with significant pharmacokinetic variability in SAH and other patient populations. This variability is mainly attributed to factors such as the severity of SAH, route of administration, and patient-specific factors including plasma protein concentration, age, renal function, liver function, and metabolism. The latter, metabolism, is influenced by genetic polymorphisms and drug-drug interactions [154].

An inverse relationship also exists between the severity of SAH, as reported by the Hunt and Hess score and the World Federation of Neurological Surgeons Grade, and the bioavailability of nimodipine [157]. Whether this phenomenon is attributable to intrinsic effects of SAH on the gastrointestinal tract or the administration route of nimodipine remains unclear.

The route of administration contributes to the known pharmacokinetic variability of nimodipine since the intake via feeding tubes has been associated with maximum concentrations mostly lower than those observed with the oral route [158–160]. The same applies to parenteral administration [158].

Nimodipine is highly bound to plasmatic alpha-acid glycoprotein (> 95%), whose levels proved to be higher in patients with SAH in the acute phase before to significantly decrease after 48 h [161]. Nimodipine concentration is also inversely proportional to the level alpha-acid glycoprotein in cerebrospinal fluid [161]. Accordingly, its distribution and clinical efficacy can be largely affected by the concentration of alpha-acid glycoprotein whose plasmatic levels are decreased in case of nephrotic syndrome, protein-losing enteropathies, or liver failure.

Patients older than 60 years have reduced first-pass metabolism of oral nimodipine compared with younger patients [162], as well as a reduced clearance [157].

Given that SAH patients also experience reduced kidney function [163, 164], the pharmacokinetics of nimodipine might be characterized by a probable lengthening of the half-life in the clinical scenario, even in patients without kidney diseases. Chronic kidney disease patients with an estimated glomerular filtration rate < 60 mL/min have a prolonged nimodipine half-life [165].

Metabolism of nimodipine is mainly by the hepatic CYP3A enzyme family [166]. The existence of a genetic polymorphism of the liver cytochrome P450 (CYP450) allowed to categorize the healthy patients in extensive, normal, intermediate, and poor metabolizers [166]. Homozygotic isoform of CYP3A5 accounts for a reduced nimodipine clearance [167], whereas patients having CYP3A5 genotype are considered poor metabolizer with consequent risk of severe bradycardia with junctional atrioventricular heart block and hypotension reported in one case [168]. Subjects affected by liver cirrhosis have been reported to have a Cmax PO 1.4- to ninefold higher than the Cmax PO observed in those with normal liver function [169].

Within the scenario of SAH also drug-to-drug interactions matter for what concern nimodipine pharmacokinetics.

The enzyme-inducing effect of the concomitant administration of anticonvulsivants as phenytoin, carbamazepine, and phenobarbital on the CYP3A enzymes has proved to decrease the nimodipine plasma concentrations and therefore its pharmacological effect [170, 171]. Conversely, no significative interactions have been reported for ranitidine, tirilazad, diazepam, propranolol, and indomethacin [172–176].

### Level of evidence for nimodipine alternative drugs

**Fig. 3:**
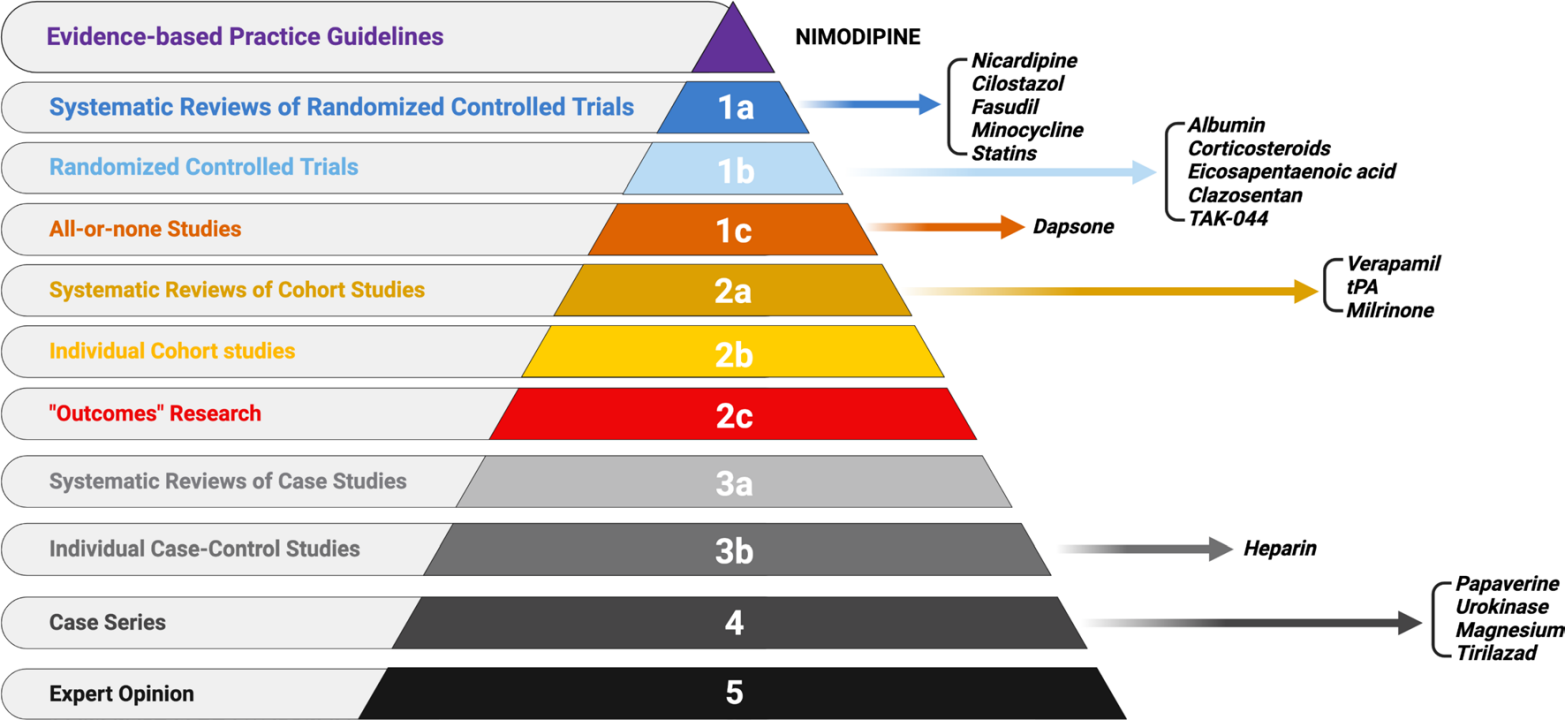
Overall level of evidence for nimodipine alternative drugs

Nicardipine, cilostazol, fasudil, minocycline, and statins reached type 1a evidence, whereas albumin, corticosteroids, eicosapentaenoic acid, clazosentan and TAK-044 reached type 1b. The role of dapsone remains interesting and the proven efficacy of verapamil, tPA, and milrinone is not to be underestimated (Fig. 3).

### Strength of recommendation for nimodipine alternative drugs

**Fig. 4:**
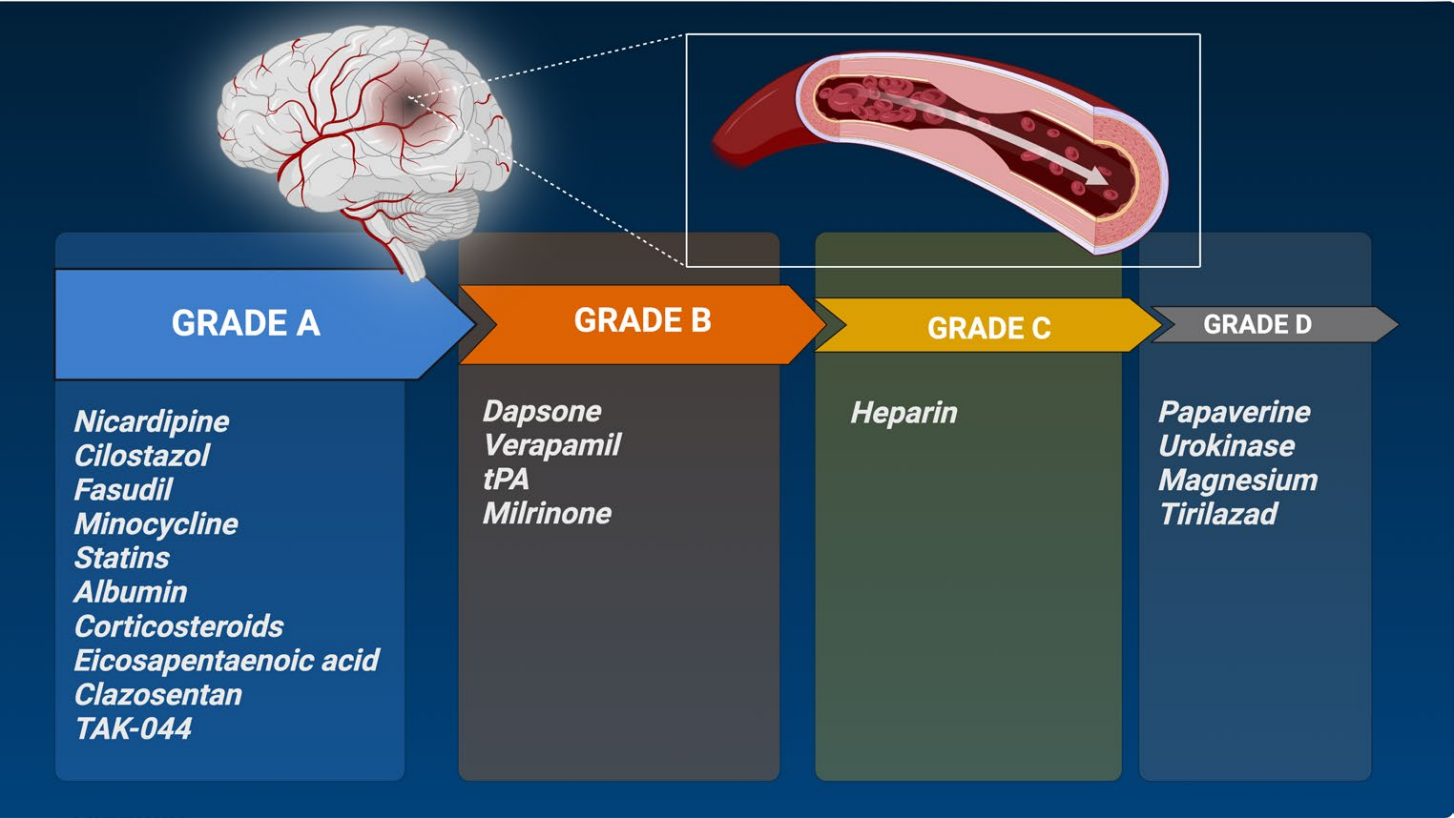
Overall strength of recommendation for nimodipine alternative drugs

Drugs with type 1a and type 1b evidence have a grade A recommendation based on their confirmed effectiveness, while dapsone, verapamil, tPA and milrinone have a grade B recommendation **(** Fig. 4**)**.

## Pharmacokinetic considerations of nimodipine alternative drugs

Within the type 1a evidence-grade A recommendation drugs, nicardipine and cilostazol have a longer half-life than nimodipine, this aspect making them theoretically advantageous in decreasing the negative effects of the blood pressure fluctuations. Similarly, eicosapentaenoic acid, dapsone and clazosentan showed an optimal pharmacokinetic profile in the type 1b evidence-grade B recommendation group. Eicosapentaenoic acid is mainly metabolized by the adrenal glands and kidneys. Accordingly, its use is more indicated in the setting of concomitant administration of phenytoin, given the latter’s induction effect on the cytochrome P-450 complex, or in poor nimodipine metabolizers [166]. Nicardipine is mainly extracted from bile, which makes it safer to use in patients with acute and chronic renal failure.

### Non-vasospasm related DCI and drugs targeting DCI

DCI is known to be responsible of delayed clinical deterioration after SAH through mechanisms other than the only cerebral vasospasm which remains, however, the most important one occurring at the level of larger extraparenchymal vessels [40, 42, 177]: They are (1) microcirculatory constriction, (2) microthrombosis, (3) cortical spreading depolarization and ischemia, and (4) neuroinflammation [149, 177].

Microcirculatory constriction consists in the disruption of cerebral autoregulation, neurovascular coupling, and blood-brain barrier, all of these aspects having detrimental effects on cerebral blood flow [178–180]. These hemodynamic effects mainly involve the distal vasculature of the supratentorial and infratentorial circles [181, 182]. The nitric oxide pathway, oxidative stress, cellular adhesion molecules, and inflammation are the main factors responsible for arteriolar vasoconstriction, while MMPs have been reported to be associated with blood-brain barrier dysfunction and cerebral edema. [180, 183]. Endothelial NO synthetase inhibitors are promising agents for the pharmacotherapy of non-vasospasm related DCI [184–186]. Further preclinical data of effectiveness came from the use of intravenous human albumin [29, 187] and erythropoietin [48, 188–190].

Microthrombosis of the distal cerebral vasculature is the final result of documented impairments in both the coagulation and fibrinolysis cascades after SAH [150, 178, 191–193]. While having a rationale, aspirin and enoxaparin failed to prove clear benefits [150]. Although effective for strictly addressing vasospasm, statins were found to be unable to decrease the rate of DCI and mortality [194]. The efficacy of the recombinant ADAMTS-13 is still limited to in-vitro observations [195, 196]. Intracisternal thrombolysis with tPA recorded some beneficial results in a meta-analysis by Amin-Hanjani et al. [61], which were largely mitigated by a phase II trial conducted by Etminan and colleagues [197].

Cortical spreading depolarization is a disorder coming from a slowly propagating depolarization wave causing amplified metabolic activity and a serious disturbance in ion homeostasis [151, 198–201]. It is correlated to arteriolar vasoconstriction, cortical spreading ischemia, and inverse neurovascular coupling ultimately leading to a glutamate-induced neurotoxic release mediated by N-methyl-D-aspartate (NMDA), alfa-amino-3-hydroxy-5-methyl-4-isoxazolepropionic acid (AMPA), and kainate receptors [151]. The rationale for using anticonvulsants for the prevention and management of DCI stems from the need to counteract epileptogenic complications. NMDA antagonists, GABA inhibitors, anesthetics, topiramate, and calcitonin gene-related peptide (CGRP) antagonists have been tested with promising preclinical data [202–204].

A robust neuroinflammation has proved to underlie both aneurysms formation [205] and aneurysmal SAH concurring in increasing the risk of DCI [149, 191, 193, 206]. This aspect accounted for the vivid interest in trying to correlate the increased levels of cerebrospinal fluid circulating inflammatory cytokines, interleukin-6, and the risk of DCI [207–210]. Neuroinflammation has also been linked to products resulting from erythrocyte disruption, with heme being one of the primary factors [211]. Interestingly, Kantor et al. demonstrated that individuals with the haptoglobin α2-α2 genotype have less protection from DCI and experience worse outcomes after aneurysmal SAH. This is due to the decreased capability of this isoform to bind hemoglobin, thereby preventing the formation of metabolites that cause inflammation [212]. Polymorphisms such as interleukin-1β -511 C > T, interleukin-6 -174G > C, and − 572G > C have been found to correlate with an increased risk of intracranial aneurysms [213, 214]. Conversely, endothelial nitric oxide VNTR A and T alleles, as well as haptoglobin 1/2 phenotypes, have shown the strongest associations with vasospasm [215, 216].

The various traditional anti-inflammatory or immunomodulatory drugs have undergone testing, yielding inconsistent outcomes. These include NSAIDs, thromboxane inhibitors, corticosteroids like methylprednisolone, cyclosporine A, complement inhibitors, statins, and monoclonal antibodies directed at cytokine receptors or cellular adhesion molecules [149, 191, 217, 218]. A long list of anti-inflammatory targets are under evaluation in preclinical models of experimental SAH; it involves MAPK (mitogen-activated protein kinase), mTOR (mechanistic target of rapamycin), Mincle/Syk (microglia macrophage-inducible C-type lectin/ spleen tyrosine kinase), PARP (poly(ADP-ribose) polymerase), RAGE (receptor for advanced glycation endproducts), S1PR (sphingosine 1-phosphate receptor) [212, 219–223].

**Fig. 5:**
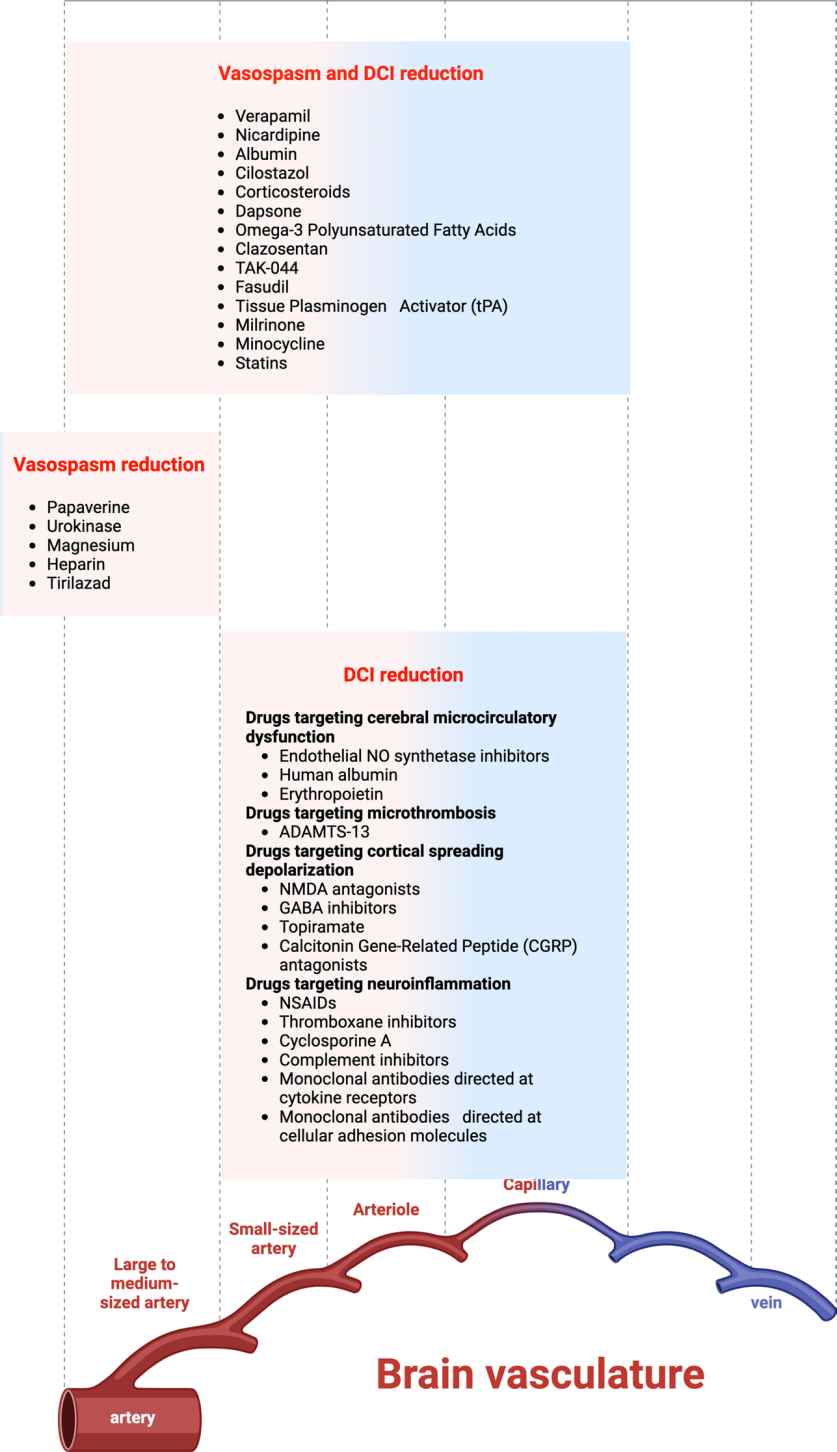
Drugs targeting vasospasm only, DCI only, and both vasospasm and DCI

Figure 5 categorizes drugs targeting vasospasm only, DCI only, and both vasospasm and DCI.

### Limitations of the study

The present review has some limitations, which can be summarized in its retrospective nature, the limited number and heterogeneity of the studies, and the intrinsic bias of the different studies involved. Interpatient pharmacokinetic variability and the potential for drug-drug interactions in patients receiving multiple therapies are further constraints.

## Conclusions

Nimodipine has the strongest evidence for reducing the incidence of radiological and clinical cerebral vasospasm and DCI, although its pharmacodynamic and pharmacokinetic shortcomings make it a suboptimal candidate even today. There was robust evidence for the efficacy and safety of nicardipine and cilostazol as valid alternatives to nimodipine, although the number of trials conducted is significantly lower than for nimodipine. Eicosapentaenoic acid, dapsone and clazosentan showed a good balance between evidence of efficacy and favorable pharmacokinetics. Combinations between different classes of drugs have been studied to a very limited extent. Aneurysmal SAH-associated vasospasm remains an area of ongoing preclinical and clinical research where the search for new drugs or associations is critical.

## Data Availability

No datasets were generated or analysed during the current study.
